# Melatonin and urological cancers: a new therapeutic approach

**DOI:** 10.1186/s12935-020-01531-1

**Published:** 2020-09-10

**Authors:** Mohammad Hossein Mehrzadi, Azam Hosseinzadeh, Kobra Bahrampour Juybari, Saeed Mehrzadi

**Affiliations:** 1 Research Center for Biochemistry and Nutrition in Metabolic Diseases, Kashan University of Medical Sciences, Kashan, Iran; 2 Razi Drug Research Center, Iran University of Medical Sciences, Tehran, Iran; 3 Department of Pharmacology, School of Medicine, Semnan University of Medical Sciences, Semnan, Iran

**Keywords:** Bladder cancer, Prostate cancer, Renal cell carcinoma, Melatonin, Chemotherapy, Radiotherapy, Urological cancers, Angiogenesis, Apoptosis, Autophagy, Oxidative stress, Inflammation, metastasis

## Abstract

Urological cancers are responsible for thousands of cancer-related deaths around the world. Despite all developments in therapeutic approaches for cancer therapy, the absence of efficient treatments is a critical and vital problematic issue for physicians and researchers. Furthermore, routine medical therapies contribute to several undesirable adverse events for patients, reducing life quality and survival time. Therefore, many attempts are needed to explore potent alternative or complementary treatments for great outcomes. Melatonin has multiple beneficial potential effects, including anticancer properties. Melatonin in combination with chemoradiation therapy or even alone could suppress urological cancers through affecting essential cellular pathways. This review discusses current evidence reporting the beneficial effect of melatonin in urological malignancies, including prostate cancer, bladder cancer, and renal cancer.

## Introduction

Urological cancers, which account for 12% of malignancy-associated deaths across the world, mostly include cancers of the prostate, bladder and kidney. Prostate cancer is the most prevalent and accounts for one million new cases, and 300,000 deaths each year [1, 2]. The second most frequent urological cancer is bladder cancer, which is the ninth most common cancer in the world. Annually, approximately 330,000 people are diagnosed with bladder cancer, which leads to about 130,000 deaths [2]. The main risk factors for bladder cancer are chronic irritation, environmental and chemical exposures (especially cigarette smoking), molecular aberrations (particularly p21 and Ras proteins and *RB1*, *TP53*, *EGFR* and *TP63* genes), and 50–70 years of age [3]. Renal cell carcinoma (RCC) possesses the greatest mortality rates, one-third of affected subjects. The main risk factors for RCC are hypertension, obesity, cigarette smoking, and age (50–70 years) [4]. Because of the resistance to treatment and metastasis, exploring novel therapeutic methods is vital for urological cancer therapy.

Melatonin is a molecule which has a broad spectrum of biological effects, including anti-angiogenic [5], anti-oxidant [6, 7], anti-inflammatory [8, 9], anti-nociceptive [10], weight-reducing, anti-obesogenic [11], anti-migration, anti-invasion [12–14], anticancer [15, 16], immunomodulatory [17], pro-apoptotic [18], and anti-proliferation activities [19]. Melatonin synchronizes circadian rhythms, and ameliorates the quality, duration and onset of sleep. Decline in the melatonin serum level, occurred during aging, various disease or artificial light exposure at night, leads to the disruption of cellular circadian rhythm; this is associated with the alterations in sleep-activity pattern, suppression of melatonin production, and deregulation of expression patterns of cancer-related genes [20, 21]. Many clinical investigations have reported melatonin beneficial application in the therapy of cancers [22]. Melatonin suppresses tumor invasion through inhibiting CCL24 via blocking the JNK pathway in osteosarcoma [13]. Melatonin represses colon cancer stem cells through modulating cellular prion protein/Oct4 axis [23], and increases brain cancer stem cell sensitivity to paclitaxel [24]. In this review, we discuss available data of melatonin therapeutic effects in the treatment of urological cancers, based on molecular signaling pathways.

## Anticancer potentials of melatonin: mechanisms of actions

Inhibitory impacts of melatonin on metastasis and growth of cancer cells have been widely studied. Of note, melatonin actions between healthy and tumoral cells are clearly different [12, 13, 25]; melatonin exerts its pro-apoptotic effects on cancer cells [26], but shows its anti-apoptotic properties in healthy cells [27]. This effect results from the differences of cancer cells with healthy cells in many ways including metabolism, gene regulation, and stress responses [28, 29]. The ability of melatonin to scavenge free radicals has been proposed by several investigations [30]; however, a few in vitro studies have reported the stimulatory effect of melatonin on ROS production at pharmacological concentrations [28]. Melatonin suppresses tumor proliferation through inhibiting telomerase activity and cell cycle kinetics [12, 31]. Melatonin exerts angiostatic features through suppressing the expression and activation of vascular endothelial growth factor (VEGF) receptor 2 and inhibiting invasion, migration, and tube formation of endothelial cells [32]. Autophagy is another important mechanism implicated in controlling cellular homeostasis [33–35]. Autophagy possesses pro-survival actions; however, excessive autophagy probably results in cell fate, a process morphologically different from apoptosis [36]. Furthermore, autophagy-deficient malignant cells succumb to radiotherapy and chemotherapy, in vivo [37]. Melatonin mediates the generation of intracellular reactive oxygen species (ROS), whose accumulation has upstream roles in mitochondria-induced autophagy and apoptosis [38]. Melatonin impairs the apoptosis resistance and proliferation of cancer cells through inactivation of ROS-induced Akt signaling pathway; Akt stimulates the up-regulation of anti-apoptotic proteins including Bcl-2, PCNA and cyclin D1 and down-regulation apoptotic proteins such as Bax. Melatonin also inhibits the invasion and migration of cancer cells *via* inhibiting ROS-activated Akt signaling, leading to the Vimentin and Snail enhancement, and E-cadherin reduction [39]. Melatonin reduces proliferation and induces apoptosis in cancer cells through regulating PI3K/AKT/mTOR, Apaf-1/caspase-9, PI3K/Akt, p300/nuclear factor kappa B (NF-κB) and COX-2/PGE2 signaling pathways [40, 41]. Mitophagy removes injured mitochondria, which impairs chemotherapy-induced mitochondrial apoptosis. Melatonin is able to sensitize cancer cells to cisplatin-mediated apoptosis via suppression of JNK/Parkin/mitophagy pathway [42]. Importantly, melatonin modulates inflammatory and angiogenic proteins which are responsible for tumor progression [43]. The nuclear translocation of NF-κB and the expression of pro-inflammatory factors, such as tumor necrosis factor-α (TNF-α), interleukin-1β (IL-1β), and IL-6 are reduced by melatonin [44]. Furthermore, melatonin reverses chemotherapy resistance through repressing the Wnt/β-catenin pathway and controls migration and invasion of cancer stem cells [45, 46]. Results from clinical studies indicate that melatonin improves the sleep and quality of life in patients with cancer. Furthermore, combination of melatonin with anticancer drugs enhances the therapeutic effect of chemotherapeutic agents and survival of patients with cancer [47, 48]. Melatonin is suggested to overcome drug resistance through (I) increasing response to chemotherapeutics agents via modulation of the expression and phosphorylation of their targets, (II) reducing the clearance of chemotherapeutics by impressing their metabolism and transport, (III) decreasing the survival of malignant cells via alteration of DNA and (IV) regulating cell death-associated mechanisms such as apoptosis and autophagy [49]. Regarding what briefly discussed above, anticancer effects of melatonin widely investigated during last decades. Herein, the therapeutic actions of melatonin have been evaluated on the pathogenesis of urological cancers.

## Therapeutic application of melatonin and urological cancers: focus on signaling pathways therapeutic application of melatonin and urological cancers: focus on signaling pathways

### Prostate cancer

The incidence of prostate cancer significantly elevates among males by increasing age. A systematic review of epidemiologic studies has reported an association between circadian disruption or sleep loss and prostate cancer [50]. A prospective association between first morning-void urinary 6-sulfatoxymelatonin (aMT6s) level and risk for prostate cancer has been reported by a case-cohort study; men with morning urinary aMT6s level below the median possess a fourfold higher risk for advanced or lethal prostate cancer compared to men with higher level [51]. Examination of circadian rhythms of melatonin showed that the level of melatonin reduces in the serum of patients with primary prostate cancer; this depression of serum melatonin has been reported to be due to a reduced pineal activity and be not caused by an enhanced metabolic degradation in the liver [52]. Melatonin increases the survival of animals by 33% when administered at the beginning or at advanced tumor stages [53]. Melatonin controls and represses this type of cancer by induction of apoptosis through regulating the generation of ROS, mitochondrial bioenergetics and several signaling pathways, including JNK and p38 pathways [54, 55].(. Melatonin considerably inhibits the expression and activity of Sirt1 protein in prostate cancer cells, which this is accompanied by a remarkable reduction in the proliferative activity of cancer cells. Prostate cancer cells are protected from anti-proliferative effects of melatonin by forced Sirt1 overexpression, proposing that Sirt1 may be a direct melatonin target [56]. The beneficial effects of melatonin in declining tumor growth are related to the reduction of angiogenesis [57]; to suppress tumor angiogenesis, melatonin inhibits the activity of hypoxia-inducible factor (HIF)-1α resulting in the inhibition of its target genes expressions in prostate cancer cells [58]. Up-regulation of miRNA-374b and miRNA-3195 by melatonin results in the attenuation of HIF-1/2 α and VEGF expression [59]. Tai and colleagues selected 120 newly diagnosed prostate cancer subjects as well as 240 age-matched controls and measured their main urine metabolites. Individuals having a high melatonin-sulfate/cortisol (MT/C) ratio or high levels of melatonin-sulfate were less probable to possess prostate cancer or malignancy in advanced stages [60].

**Fig. 1:**
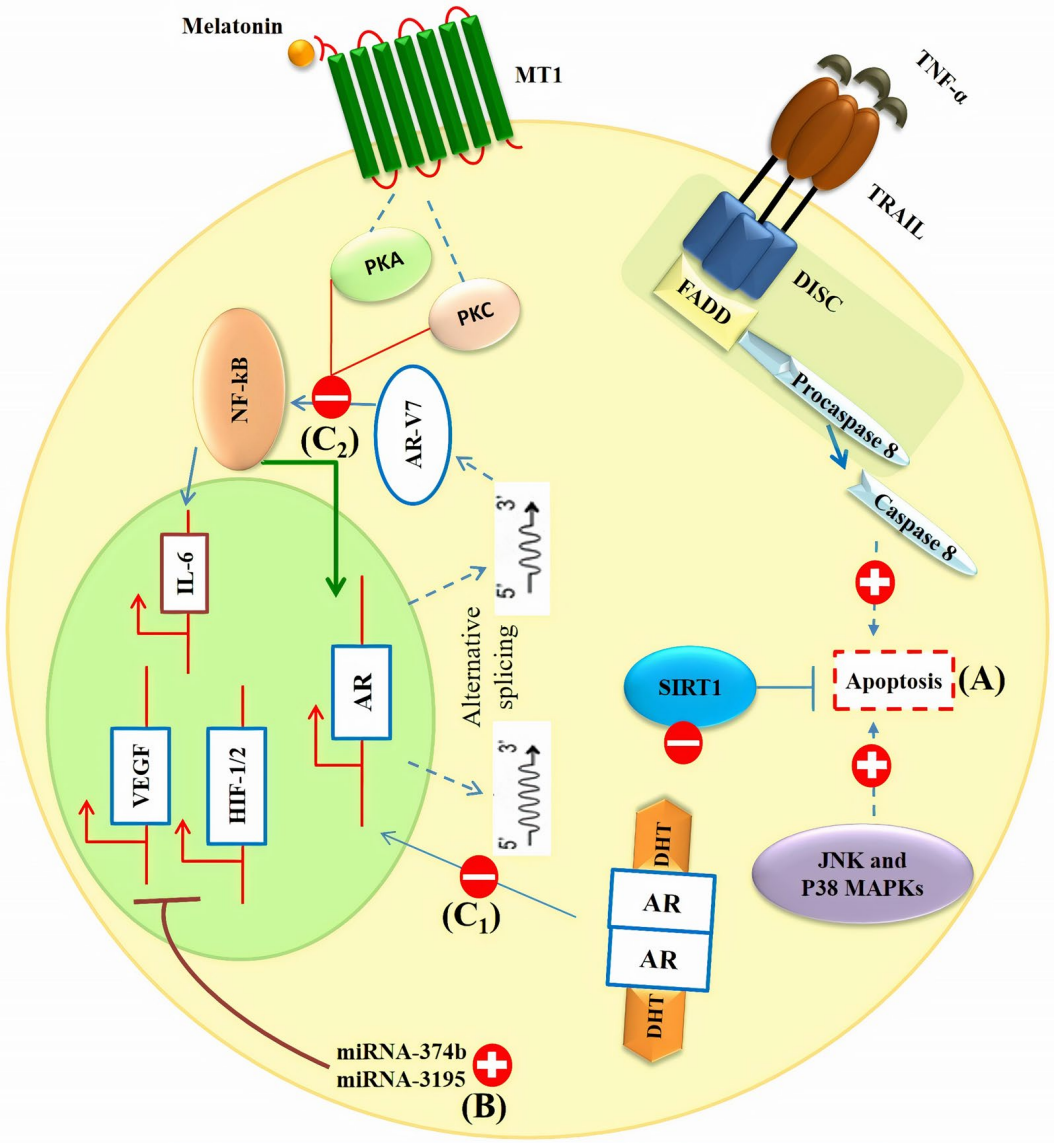
Melatonin (**a**) promotes apoptosis in prostate cancer cells through activating TNF-α/TRAIL, JNK and P38 signaling pathways, and inhibiting SIRT1 pathway, (**b**) inhibits angiogenesis by inhibition of HIF-1/2α and VEGF expression and **(C**_**1**_**)** exerts anti-androgenic effects by inhibiting nuclear translocation of androgen receptor and **(C**_**2**_**)** MT1 receptor-dependent disruption of positive interaction between androgen receptor splice variant-7 (AR-V7) expression and NF-κB/IL-6 signaling

Melatonin exerts anti-androgenic effects on prostate cells through blocking androgen receptor nuclear translocation and disrupting the positive interaction between androgen receptor splice variant-7 (AR-V7) expression and activated NF-κB/IL-6 signaling [53, 61]. This anti-androgenic effect of melatonin is mediated by the activation of MT1 receptor leading to the delay in the development of castration resistance in advanced prostate cancer [61]. Melatonin promotes cell toxicity and death caused by cytokines including TNF-α and TNF-related apoptosis-inducing ligand (TRAIL) without affecting the action of chemotherapeutic agents [62]. Terraneo et al. investigated the effect of melatonin on prostate cancer cells when delivered by cryopass-laser or intraperitoneal administration. Intraperitoneal administration of melatonin has been reported to be as effective as cryopass-laser therapy in attenuating prostate cancer cell growth, and influencing redox balance and melatonin plasma level. The effect of cryopass-laser is less than intraperitoneal delivery route of melatonin in enhancing Nrf2 expression and melatonin content in tumor mass. However, cryopass-laser treatment of melatonin is as effective as its intraperitoneal administration in the inhibition of HIF-1α. Overall, cryopass-laser therapy could be an effective method to transdermal delivery of melatonin to the site of action without causing pain [63]. To prove melatonin effectiveness in prostate cancer therapy, further studies are needed. Table 1; Fig. 1 summarize present information of melatonin therapy for prostate cancer.

**Table 1:** Investigations on melatonin treatment against prostate cancer

Melatonin dose or concentration	Targets	Effects	Model	Cell line	Refs
1 pM, 1 nM, 1 μM, 1 mM	mTOR, ERK1/2, Akt, OXPHOS, ROS	Anti-proliferative and antioxidant effects	In vitro	PNT1A	[55]
UCM 1037 (analogue)	Androgen receptor, Akt	Anti-proliferative and cytotoxic effects against cancer cells	In vitro	LNCaP, PC3, DU145, 22Rv1	[76]
1 mM	Pentose phosphate pathway	Decreased LDH activity, tricarboxylic acid cycle, ATP/AMP ratio, glucose uptake, and lactate labeling Limited glycolysis	In vitro	LNCaP , PC-3	[77]
3 mg/kg	Nrf2, HIF-1α	Inhibited tumor growth	In vivo	LNCaP	[63]
10^− 6^ M	NF-κB, AR-V7, IL-6,	Delayed castration resistance development	In vitro	LNCaP, 22Rv1	[61]
200 µg/ml 50 µM–1 mM	MAPK/ERK, IGFBP3	Increased survival time of TRAMP mice when administered at the initiation or advanced stages	In vivo, in vitro	LNCaP	[53]
10 µg/kg 500 µM, 5 mM, 10 mM	Androgen receptor (AR), PCNA, MTR1B	proliferative and anti-apoptotic effects in prostate cells subjected to HG levels	In vivo, in vitro	PNTA1, PC-3	[78]
1 mM	VEGF, HIF-1α, HIF-2α, miR-3195, miR-374b	Anti-angiogenic activity	In vitro	PC-3	[59]
1 mg/kg	Nrf2, Ki67, HIF-1α, Akt	Inhibited cancer growth and exerted anti-angiogenic effects	In vivo	LNCaP	[57]
10^− 8^ M	p27, NF-κB, MT1,	Anti-proliferative effects	In vitro	LNCaP, 22Rv1	[79]
1 mM	TRAIL, TNF-α	Promotes cell toxicity and cancer cell death, inhibited oxidative stress, and suppressed cancer cell proliferation	In vitro	LNCaP, PC-3	[62]
10 mg/kg	GSH, MDA, SOD	Inhibited tumor growth and oxidative stress	In vivo	–	[80]
10^− 11^-10^− 5^ M	MT1, p27, AR	Anti-proliferative effects	In vitro	RWPE-1, 22Rv1, VCaP, LNCaP	[81]
1 mM	Akt/GSK-3β, HIF-1α, SPHK1, VEGF, von Hippel-Lindau	Antioxidant effects	In vitro	PC-3	[82]
100 nM–2 mM	Sirt1, IGF-1)/IGFBP3, PCNA, Ki-67	Anti-proliferative effects Inhibited tumorigenesis	In vivo, in vitro	PC-3, DU145, 22Rν1, LNCaP	[56]
100 µM, 1 mM, 2mM	Per2, Clock, Bmal1	Anti-proliferative effects Caused a resynchronization of oscillatory circadian rhythm genes	In vitro	PC-3, DU145, 22Rν1, LNCaP	[83]
10^− 8^-10^− 3^ M	–	Inhibited viability and induced apoptosis	In vitro	PC-3, DU145, 22Rν1, LNCaP	[84]
1 mM	HIF-1α,	Anti-angiogenic effect	In vitro	PC-3, DU145, LNCaP	[58]
0–3 mM	p38, JNK	Induced apoptosis Inhibited cancer cell growth	In vitro	LNCaP	[54]
10^− 9^, 10^− 8^, 10^− 7^	PKA, PKC, p27, MT1	Anti-proliferative effects	In vitro	22Rv1	[85]
10^− 11^, 10^− 5^	p27, PKA, PKC, MT1, androgen signaling	Anti-proliferative effects	In vitro	22Rv1	[86]
0.5, 1 mM	–	Induced cell cycle arrest and cellular differentiation Inhibited proliferation of cancer cells	In vitro	LNCaP, PC-3,	[87]
5 mg	MT1	Anti-proliferative effects Induced stabilization of patient’s hormone-refractory disease	Human	–	[88]
4 µg/g	EGF, Cyclin D1	Inhibited tumor growth and proliferation	In vivo	PC-3, DU145, LNCaP	[89]
–	–	AR activity attenuation by melatonin is not due to inhibition of AR binding to the androgen responsive element (ARE)	In vitro	LNCaP, PC-3	[90]
4 µg/g	MT1	Anti-proliferative effects	In vivo	PC-3, LNCaP	[91]
0.01–100 nM	cAMP	suppressed cancer cell proliferation and induced cell cycle arrest	In vitro	DU145	[92]
5 × 10^−11^-5 × 10 ^−5^	MT1, sex steroid-mediated calcium influx	Anti-proliferative effects	In vitro	LNCaP	[93]
0.01–1000 nM	Mel1a receptor	Anti-proliferative effects	In vitro	LNCaP	[94]
20 mg	IGF-1, PRL	Combination therapy with triptorelin and melatonin decreased PSA mean concentrations Melatonin reversed clinical resistance to LHRH analogue triptorelin in metastatic prostate cancer	Human	–	[95]
50 µg	–	Inhibited tumor growth	In vivo	Dunning *R*-*3327*-*HIF* tumor	[96]

### Bladder cancer

**Fig. 2:**
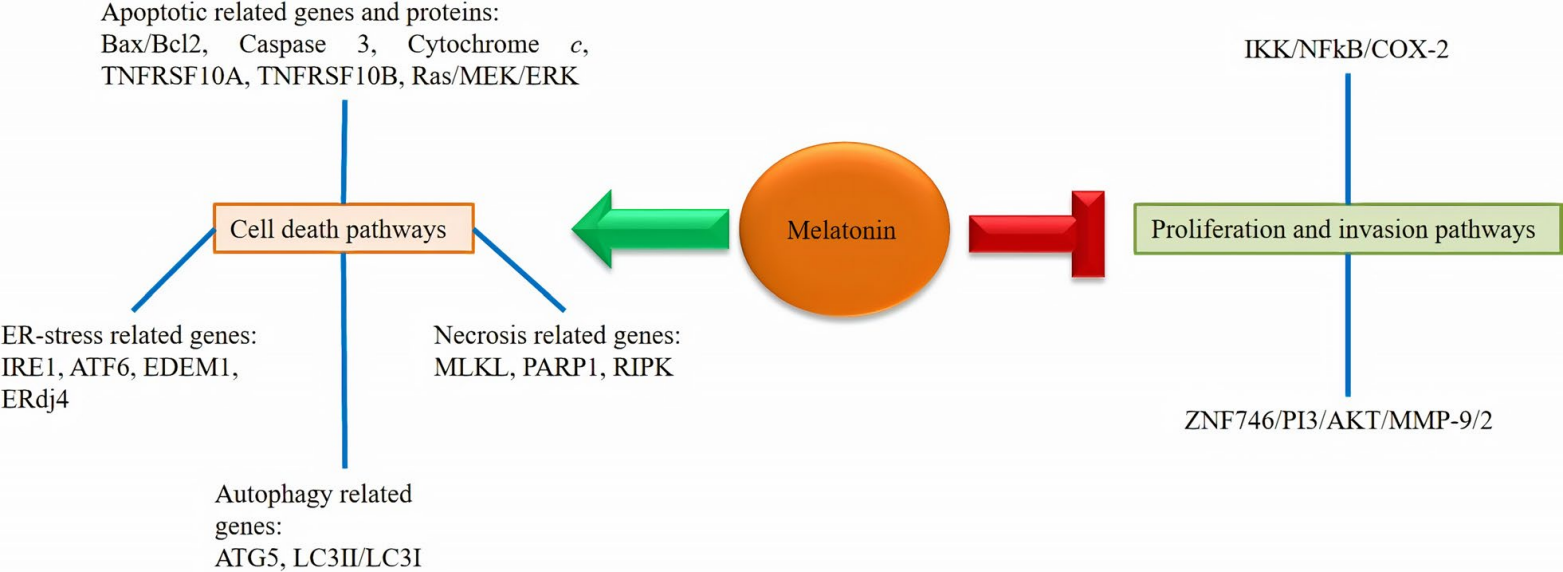
Melatonin affects some signaling pathways leading to the induction of cell death and inhibition of invasion and proliferation of bladder cancer cells

Radical cystectomy is known as the standard therapy for bladder cancer with neoadjuvant chemotherapy [64]; however, 5-year survival of subjects with metastatic form of this cancer is still low [65, 66]. Although various therapeutic approaches have been developed up to now, bladder cancer mortality rate has not significantly ameliorated. Therefore, finding novel effective therapies are required. As discussed in detail, melatonin deserves to be chosen at least as an adjuvant for the therapy of diverse cancers. Few but valuable studies have investigated the effect of melatonin on bladder cancer. Therefore, we summarize them here and in Table 2; Fig. 2.

**Table 2:** Results from experimental studies of melatonin application against bladder cancer

Melatonin dose or concentration	Targets	Effects	Model	Cell line	Refs
10 mg/kg 1 mM	cytochrome c, NF-κB, COX-2, IKKβ	Combination of melatonin and curcumin induced cell apoptosis Melatonin exerted pro-apoptotic, anti-migration, and anti-proliferative functions Melatonin synergized curcumin ability to suppress tumor growth	In vivo, in vitro	5637, UMUC3, T24	[67]
100 mg/kg	ZNF746 , p-AKT/MMP-2/MMP-9	Inhibited cancer cell growth, invasion, and migration Induced cell cycle arrest Suppressed oxidative stress	In vivo, in vitro	HT1376, HT1197, RT4, T24	[68]
10^− 6^ m	Wnt, E-cadherin, N-cadherin Raf/MEK/ERK	Combination of valproic acid and melatonin enhanced cytotoxicity by modulating cell death pathways	In vitro	UC3	[69]

Synergistic anticancer effects of melatonin in combination with curcumin have been evaluated against bladder cancer [67]; this combination results in a promoted suppression of bladder cancer cell proliferation. Moreover, melatonin and curcumin combination blocks the activity of IκB kinase β (IKKβ), leading to the repression of NF-κB nuclear translocation and their binding on COX-2 promoter. This combination mediates apoptosis in bladder cancer cells *via* increasing cytochrome c release into the cytosol. Therefore, melatonin synergizes curcumin suppressive impacts against bladder cancer growth through promoting the pro-apoptotic, anti-migration, and anti-proliferation functions. This indicates that this combination might reveal efficient therapeutic potential in the therapy of bladder cancer. Melatonin induces cell cycle arrest at G0 phase and inhibits colony formation, mitochondrial membrane potential, cell migration, and the growth of bladder cancer cells. Melatonin also blocks oxidative stress, and inhibits AKT-MMP9 signaling pathway leading to the reduction of invasion, migration, and growth of bladder cancer cells [68].

Combination of valproic acid and melatonin stimulates the expression of particular genes involved in necrosis (RIPK1, PARP-1, and MLKL), autophagy (ATG5, ATG3, and BECN) and apoptosis (such as TNFRSF10B and TNFRSF10A). This combination activates Raf/MEK/ERK and Wnt signaling pathways, up-regulates expressions of E-cadherin and endoplasmic reticulum-stress-related genes including ERdj4, EDEM1, IRE1, and ATF6 and down-regulates expressions of Slug, Snail, Fibronectin, and *N*-cadherin. These suggest that combination of valproic acid and melatonin increases cytotoxicity through modulating cell death pathways in bladder cancer [69].

### Kidney cancer

Kidney cancer is responsible for 2–3% of all cancers, and RCC is the most common type of this cancer. Among urological malignancies, RCC is believed to be the most lethal [70]. The 5-year survival rate of RCC is approximately 93%; however, this rate for patients with metastatic RCC is 12% [71]. Similar to other urological cancers, searching for appropriate therapy for the treatment of this malignancy is essential. Melatonin is believed to possess the potential to suppress this cancer.

**Fig. 3:**
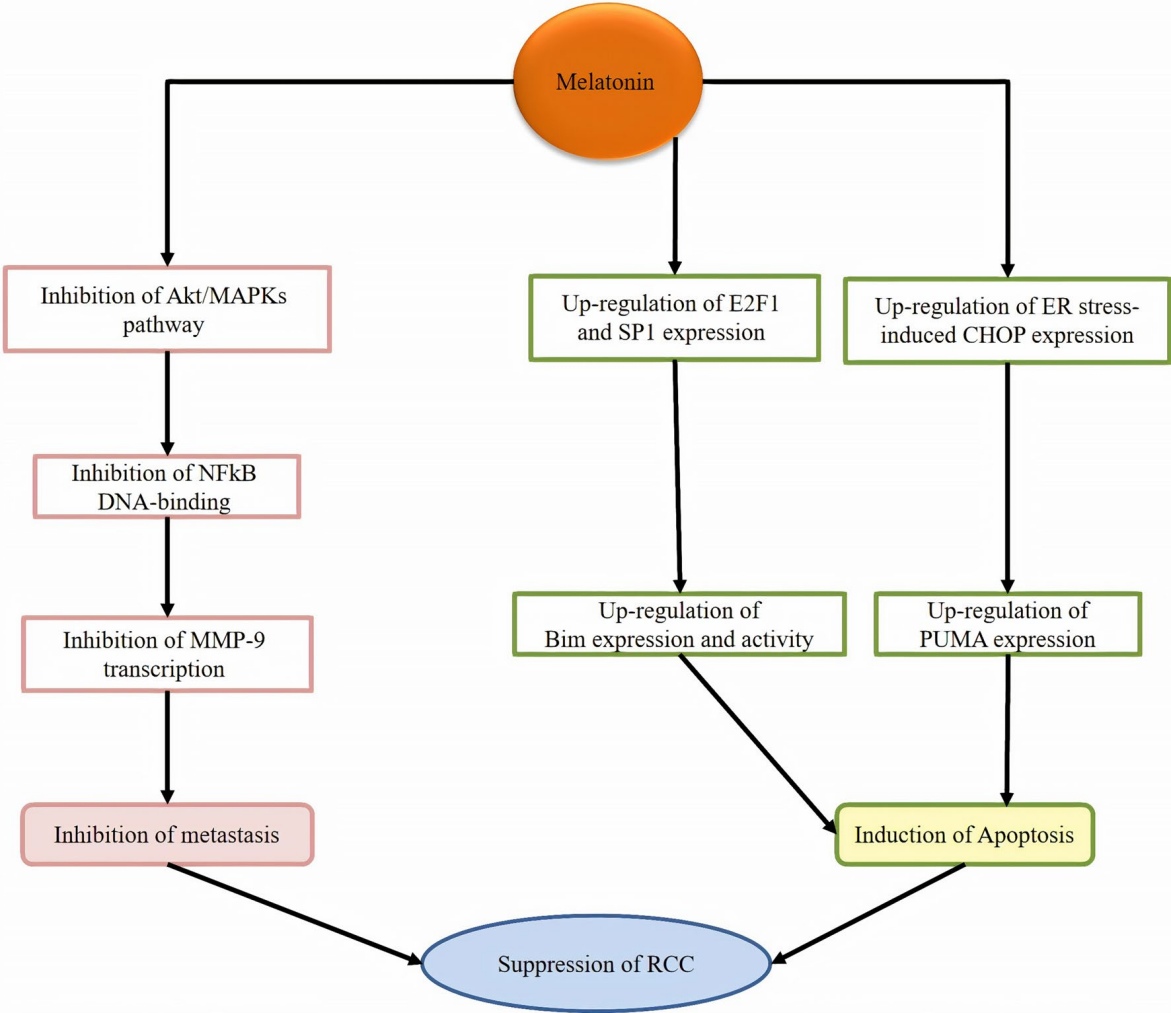
Melatonin suppresses RCC through affecting signaling molecules involved in metastasis and apoptosis

Melatonin suppresses RCC metastasis by suppressing Akt-MAPKs pathway, DNA-binding activity of NF-κB and MMP-9 transactivation [72]. Combination of melatonin and thapsigargin induces apoptosis in renal cancer cells through up-regulating CCAAT-enhancer-binding proteins homologous protein (CHOP) expression; the up-regulation of CHOP expression is melatonin receptor-independent and may result from antioxidant properties of melatonin [73]. Furthermore, Kahweol and melatonin combination up-regulates the p53-upregulated modulator of apoptosis (PUMA) through endoplasmic reticulum stress-induced CHOP induction and p53-independent pathway [74]. Melatonin could induce apoptosis in renal cancer cells through up-regulating the expression of E2F1 and Sp1, leading to the enhancement of the expression of Bcl-2-interacting mediator of cell death (Bim). Melatonin also modulates the stability of Bim protein *via* inhibiting proteasome activities. However, up-regulation of Bim induced by melatonin is independent of melatonin receptors and antioxidant potentials. Overall, these findings show that melatonin mediates apoptosis by up-regulating the expression of Bim at transcriptional levels and at the post-translational levels [75]. Table 3; Fig. 3 illustrates a summary of carried out investigations related to melatonin therapeutic roles in renal cancer therapy.

**Table 3:** A summary of current findings of melatonin for renal cancer treatment

Melatonin dose or concentration	Targets	Effects	Model	Cell line	Refs
200 mg/kg 0.5, 1, 2 µmol/L	PGC1A, UCP1	Eliminated the abnormal lipid deposits Repressed tumor progression Induced autophagy	In vivo	HK2, 786-O, A498, Caki‐1, ACHN	[97]
0.5–2 mM	MMP-9, JNK1/2, ERK1/2, MT1	Suppressed metastasis and invasion	In vitro	Caki-1, Achn	[72]
0.1, 0.5, or 1 mM	Bim, E2F1, Sp1, proteasome	Induced apoptosis	In vitro	A549, HT29, Caki	[75]
20 mg/kg 10 µM	HIF-1α	Inhibits tumor growth and blocks tumor angiogenesis	In vivo, in vitro	RENCA	[98]
1 mM	CHOP	Induced apoptosis	In vitro	HCT116, HT29, Caki	[73]
1 mM	PUMA	Induced apoptosis	In vitro	Caki	[74]
1 mM	Mcl-1	Attenuated oxaliplatin-mediated apoptosis	In vitro	Caki	[99]
20 mg	–	Increased survival Abrogated the negative influences of opioids on IL-2 immunotherapy cancer cells	Human	–	[100]
40 mg	–	Combination of immunotherapy with IL-2 plus melatonin increased survival time, and lymphocyte and eosinophil number	Human	–	[101]
10 mg	–	In addition to anticancer effects, low doses of human lymphoblastoid interferon and melatonin showed no toxicity in patients	Human	−	[102]

## Conclusion

Urological cancers are serious life-threatening diseases for societies and account for thousands of morbidity and mortality each year. Because standard therapies have not been able to increase survival time in affected patients, researchers should make so many attempts to produce further efficient drugs as alternative, complementary or adjuvant for cancer therapy. In addition to the improvement of sleep and quality of life in patients with cancer, melatonin in combination with anticancer agents increases the efficacy of routine medicine and survival rate of patients with cancer. The present review suggests that melatonin can be utilized as adjuvant of cancer therapies through reducing possible adverse events of chemotherapy or radiotherapy and increasing the sensitivity of cancer cells to medical treatments. The underlying mechanisms mainly include inhibition of cell survival, metastasis, angiogenesis, and clone formation. Furthermore, melatonin reduces resistance to treatment in cancers through the modulation of the expression and phosphorylation of drug targets, the reduction of the clearance of chemotherapeutics, the alteration of DNA of malignant cells and the regulation of cell death-associated mechanisms such as apoptosis and autophagy. Notably, it is obvious that multiple studies should be conducted, particularly human trials, to prove its safety and effectiveness in patients with different malignancies, including urological cancers.

## Data Availability

Not applicable.

## Abbreviations

RCC: Renal cell carcinoma
CCL24: C-C motif chemokine ligand 24
JNK: c-Jun N-terminal kinase
Oct4: Octamer-binding transcription factor 4
ROS: Reactive oxygen species
VEGF: Vascular endothelial growth factor
TNF-α: Tumor necrosis factor-α
IL-1β: Interleukin-1β
IL-6: Interleukin-6
Nrf2: Nuclear factor erythroid 2-related factor 2
Apaf-1: Apoptotic protease activating factor-1
COX-2: Cyclooxygenase-2
PGE2: Prostaglandin E2
Sirt1: Sirtuin
PC-3 cells: Human prostate cancer cells
HIF: Hypoxia-inducible factor
MT/C: Melatonin-sulfate/cortisol
TRAIL: TNF-related apoptosis-inducing ligand
IKKβ: IκB kinase β
MMP9: Matrix metallopeptidase 9
RIPK1: Receptor-interacting serine/threonine-protein kinase 1
PARP-1: Poly [ADP-ribose] polymerase 1
MLKL: Mixed lineage kinase domain like pseudokinase
ATG: Autophagy related
ERdj4: Endoplasmic reticulum localized DnaJ 4
EDEM1: Endoplasmic reticulum degradation enhancing alpha-mannosidase like protein 1
IRE1: Inositol-requiring enzyme 1
ATF6: Activating transcription factor 6
ERK: Extracellular signal-regulated kinase
Raf: Raf-1 proto-oncogene, serine/threonine kinase
MEK: Mitogen-activated protein kinase kinase
MAPK: Mitogen-activated protein kinase
CHOP: CCAAT-enhancer-binding proteins homologous protein
PUMA: p53-upregulated modulator of apoptosis
GSH: Glutathione
Sp1: Specificity protein 1
Bim: Bcl-2-interacting mediator of cell death
PI3K: Phosphatidylinositol-3-kinase
Akt: Protein kinase B
