# Correction to: Melatonin and urological cancers: a new therapeutic approach

**DOI:** 10.1186/s12935-020-01564-6

**Published:** 2020-09-28

**Authors:** Mohammad Hossein Pourhanifeh, Azam Hosseinzadeh, Kobra Bahrampour Juybari, Saeed Mehrzadi

**Affiliations:** 1 Research Center for Biochemistry and Nutrition in Metabolic Diseases, Kashan University of Medical Sciences, Kashan, Iran; 2 Razi Drug Research, Center, Iran University of Medical Sciences, Tehran, Iran; 3 Department of Pharmacology, School of Medicine, Semnan University of Medical Sciences, Semnan, Iran

## Correction to: Cancer Cell Int 20:444 (2020) 10.1186/s12935-020-01531-1

Following publication of the original article [1], we were notified of a mistake in the spelling of the first author.

- Incorrect spelling: Mohammad Hossein Mehrzadi
- Correct spelling: Mohammad Hossein Pourhanifeh

The original article has been corrected.
